# Ultrasound-Assisted versus Endoscopic Nasojejunal Tube Placement for Acute Pancreatitis: A Retrospective Feasibility Study

**DOI:** 10.1155/2021/4903241

**Published:** 2021-10-05

**Authors:** Gang Li, Jiajia Lin, Yang Liu, Qi Yang, Zhihui Tong, Lu Ke, Weiqin Li

**Affiliations:** Center of Severe Acute Pancreatitis (CSAP), Department of Critical Care Medicine, Jinling Hospital, Medical School of Nanjing University, No. 305 Zhongshan East Road, Nanjing, 210002 Jiangsu, China

## Abstract

**Objective:**

The optimal technique for nasojejunal tube (NJT) placement in terms of facilitating early enteral nutrition (EN) in patients with acute pancreatitis (AP) is unclear. In this study, we aimed to evaluate the impact of two common techniques on EN implementation and clinical outcomes in a group of AP patients.

**Methods:**

This is a retrospective study. All the data were extracted from an electronic database from August 2015 to October 2017. Patients with a diagnosis of AP requiring NJT placement were retrospectively analyzed. The primary outcome was the successful procedural rate.

**Results:**

A total of 53 eligible patients were enrolled, of whom 30 received an ultrasound-assisted technique and the rest received the endoscopy method (*n* = 23). There was no difference in success rates of initial placement procedures between the two groups (93.3% and 95.7% in the ultrasound-assisted group and endoscopy group, respectively). The mean amount of EN delivery within the first three days after NJT placement was significantly higher in the ultrasound-assisted group (841.4 kcal (95% CI: 738.8, 944 kcal) vs. 652.5 kcal (95% CI: 562.5, 742.6 kcal), *P* = 0.018). Moreover, a slight increased postprocedural intra-abdominal pressure (IAP) was observed in patients undergoing endoscopic procedures, but not in the ultrasound-assisted group, especially at 6 hours after NJT placement (0.35 vs. -2.01 from baseline, *P* < 0.05). For clinical outcomes, we observed no difference between groups.

**Conclusion:**

Compared with endoscopic procedures, ultrasound-assisted NJT placement possesses the acceptable success rates of initial placement procedures.

## 1. Introduction

Enteral nutrition (EN) is one of the cornerstones for the management of acute pancreatitis (AP) [1–3]. Nasojejunal feeding is necessary for EN implementation in a group of critically ill patients with a high risk of aspiration or intolerance to gastric feeding [4, 5], like those with high intra-abdominal pressure (IAP) [6]. Placing NJT beyond the ligament of Treitz potentially reduces the risk of gastroesophageal reflux, thereby avoiding aspiration.

There are several techniques available for placing NJT [7–12]. The blind method is the most convenient technique and is frequently used. However, it is associated with considerable failure rates and may lead to complications such as pneumothorax and pneumonia due to inadvertent placement into the bronchus [13]. The fluoroscopic technique is reliable but costly, often requiring patient transfer and delaying early initiation of feeding [14]. Moreover, radiation exposure is not negligible when fluoroscopic guidance is used [15]. The endoscopic approach has become popular in recent years because of its high success rate and safety profile. However, sedative and air insufflation are unavoidable in endoscopic procedures, which could potentially aggravate gastrointestinal dysmotility, thereby disturbing EN implementation [16]. Ultrasound is a noninvasive method and widely available in modern ICUs. A previous study showed that bedside real-time ultrasound-assisted placement is convenient and safe for AP patients with a high success rate [17].

We conducted this study to assess the impact of ultrasound-assisted NJT placement and endoscopic NJT placement on the implementation of EN for a group of AP patients.

## 2. Methods

### 2.1. Study Population

The present study is a single-center, retrospective study conducted in the Center of Severe Acute Pancreatitis (CSAP), Jinling Hospital, which is one of the biggest referral centers for severe acute pancreatitis in China. The study period was between August 2015 and October 2017. All the data were extracted from an electronic database, which stored prospectively collected clinical data of all AP patients admitted to the CSAP since 2014. We obtained the approval of the Acute Pancreatitis Database Management Committee (2018 JLAPDMC-009), and all the analyses were performed in accordance with the committee's regulation. Informed consent involving data storage and academic use of data was obtained from each patient during their hospitalization. All patients with a primary diagnosis of AP admitted to our center, requiring NJT placement, were assessed for eligibility. The diagnosis of AP required two of the following three features: (1) abdominal pain consistent with acute pancreatitis, (2) serum lipase activity or amylase activity at least three times greater than the upper limit of normal, and (3) characteristic findings of acute pancreatitis on computed tomography [18]. The indications of introducing the NJT were as follows: (1) large gastric residual volume (GRV) > 500 ml/6 h and (2) patients with nausea, vomiting, and abdominal distention due to delay in gastric emptying, suggesting intolerance to gastric feeding. Patients were included if they are aged between 18 and 65 years old and within seven days from the onset of symptoms when receiving NJT placement. Exclusion criteria were as follows: receiving NJT placement before admission, previous anatomy-altering upper gastrointestinal surgery, gastric malignancy, esophageal varices, gastrostomy or jejunostomy, pregnancy, severe cardiac arrhythmia, and death expected within 48 h.

### 2.2. Nasojejunal Tube Placement

The method for nasojejunal tube placement was upon attending physicians' discretion. When the ultrasound-assisted technique was adopted, the nasoenteral feeding tube (CORPAK MedSystems, 10FR 55”, Buffalo Grove, Illinois) was placed by a seasoned physician, with the patient lying in a comfortable, semiupright position before the procedure. Colored Doppler ultrasound was used to confirm the position of the tip, as described in a previous study [19]. The tube was inserted along the naris and advanced approximately 40-45 cm for placement into the stomach. Meanwhile, a standard 3.5 MHz and curvilinear array probe was placed at the epigastrium and below the xiphoid to detect the tube. The feeding tube was identified by demonstrating a fine, long, and slightly hyperechoic structure. If the definite structure cannot be found, we used color Doppler to scan the area, then 5 ml normal saline (NS) was injected into the feeding tube, and the color Doppler imaging revealed the presence of the feeding tube. For patients undergoing endoscopic procedures, the NJT was inserted by an experienced gastroenterologist, with patients in a left recumbent position, if possible. A guidewire (TERUMO Radifocus®, Japan) was loaded into the feeding tube. The internal lumen of the feeding tube was flushed with water. The outer wall of the feeding tube was then lubricated and inserted through the naris into the stomach. A pair of biopsy forceps was inserted via the endoscopic biopsy channel (GIF XP-260; Olympus, Tokyo, Japan) and used to grasp the tip of the feeding tube firmly. The endoscope pulled the feeding tube through the pyloric sphincter into the distal duodenum. The biopsy forceps advanced the feeding tube as far as possible. When the endoscope was withdrawn to the stomach, the biopsy forceps were loosened and gently shaken to separate from the tube. Patients were sedated with 10 ml 0.1% propofol, and continuous infusion would be applied as needed at the anesthetist's notice. NJT placement was considered successful when an abdominal radiograph confirmed that the distal end was reaching beyond the ligament of Treitz.

### 2.3. Outcomes

The primary objective was the successful procedural rate. The secondary outcomes include the amount of EN delivered in the first 72 hours following the tube placement procedure, the time between prescription and initiation of NJT placement, the change of IAP from baseline (before the procedure) at six time points (0, 1 h, 6 h, 12 h, 18 h, and 24 h) after NJT placement, the procedural time, the length of ICU stay, the incidence of infected pancreatic necrosis (IPN), and mortality by hospital discharge or death.

### 2.4. Data Collection

Data concerning demographic and baseline clinical characteristics including age, gender, etiologies, body mass index, and clinical scores like Acute Physiology and Chronic Health Evaluation II (APACHE II) score, NRS-2002 score, CT severity index, and sequential organ failure assessment (SOFA) score at admission were extracted from the database. Energy requirements were calculated using the Harris and Benedict equation according to the ideal body weight. The time from prescription to initiation of NJT placement, levels of IAP at six time points (0, 1 h, 6 h, 12 h, 18 h, and 24 h) after NJT placement, and procedural time were obtained from procedural recordings.

### 2.5. Statistical Analysis

Analysis was performed based on the initial treatment assignment. Data were analyzed using SPSS for Windows version 18 (SPSS Inc., Chicago, IL, USA). The distribution of continuous variables was examined for normality using the Shapiro-Wilk test. Normally distributed data were expressed as mean ± SD and compared with the independent-samples *t*-test. Nonnormal distribution data were expressed as median (interquartile range) and analyzed by nonparametric tests. Categorical variables were expressed as absolute numbers (percentage) and compared by Pearson's chi-square or Fisher exact test as indicated. A difference with a two-tailed *P* < 0.05 is considered statistically significant.

## 3. Results

### 3.1. Patient Characteristics

A total of 53 eligible patients receiving NJT placement were included. Of the 53 patients who received NJT placement in our center during the study period, 12 patients had high GRV (>500 ml) and the others had intolerance symptoms with gastric feeding, such as vomiting, diarrhea, aspiration, regurgitation, and abdominal distention. The baseline characteristics of the 53 study patients are shown in Table 1. During the study period, 30 (56.7%) of the study subjects were included in the ultrasound-assisted group and 23 (43.3%) in the endoscopic group. In the ultrasound-assisted group, one patient was switched to endoscopy due to technical failure. In the endoscopy group, two patients underwent alternative ultrasound-assisted NJT placement because they suffered hypoxemia after basic anesthesia and were deemed as intolerant to the endoscopic procedure. There were no differences in baseline characteristics between the two groups. The median APACHE II score was 12.5 (8-17) in the ultrasound-assisted group and 11 (8-16) in the endoscopy group (*P* = 0.627), and analysis of the SOFA score also confirmed no significant difference between the two groups (5 in the ultrasound-assisted group compared with 3 in the endoscopy group, *P* = 0.148).

### 3.2. Clinical Outcomes

The success rates of initial placement procedures did not differ between groups (93.3% and 95.7% in the ultrasound-assisted group and endoscopy group, respectively). No severe adverse events occurred during all the procedures. As shown in Table 2, procedural duration was longer in the ultrasound-assisted group than in the endoscopic group (20 min (17.25-25) vs. 20 min (15-20), *P* = 0.02). However, the interval between prescription and initiation of NJT placement was shorter in the ultrasound-assisted group (4 (3-6) h vs. 12 (6-24) h, *P* < 0.001). For clinical outcomes, the rate of IPN, length of hospital stay, and mortality were all comparable between the two groups.

**Figure 1:**
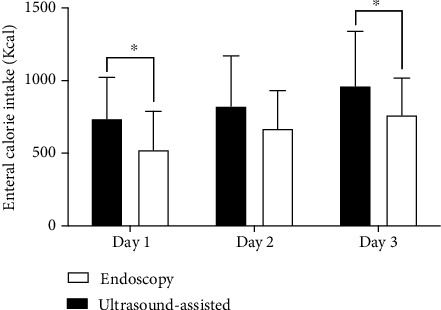
The daily amount of energy intake for the first three days after nasojejunal tube placement. All patients were divided into ultrasound-assisted (dark boxes) and endoscopy (light boxes) groups. The ultrasound-assisted group showed a much higher amount of calorie intake measured by the proportion of the target at each day (^∗^*P* < 0.05).

The total amount of EN delivery within the following 72 h after NJT placement in the ultrasound-assisted group was higher than that in the endoscopy group (841.4 kcal (95% CI: 738.8, 944 kcal) compared with 652.5 kcal (95% CI: 562.5, 742.6 kcal), *P* = 0.018). The daily amount of calorie intake was elevated progressively in both groups (Figure 1). However, enteral nutrition calories delivered were greater in the ultrasound-assisted group than in the endoscopic group on day 1 and day 3 after NJT placement (*P* < 0.05).

**Figure 2:**
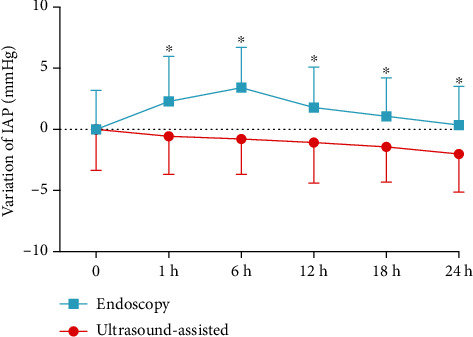
Change of intra-abdominal pressure in two groups after nasojejunal tube placement. IAP: intra-abdominal pressure (^∗^*P* < 0.05).

IAP increased from the baseline in the endoscopic group after the placement procedures, while there was a downward trend observed in the ultrasound-assisted group. The discrepancy was significant at six time points (0, 1 h, 6 h, 12 h, 18 h, and 24 h) after NJT placement (Figure 2).

## 4. Discussion

The techniques for placing NJT vary in different centers, but the comparison between the ultrasound and endoscopy was seldom studied. Our results demonstrated that the ultrasound-assisted bedside approach was comparable to the endoscopic technique with similar success rates of initial placement procedures.

Early enteral nutrition plays a vital role in the management of AP not only from a nutritional perspective but also because of the beneficial effect on mucosal integrity and function it provides, which could potentially help prevent bacterial translocation and atrophy of lymphoid tissue associated with the gut, in this case potentially reducing the incidence of infectious complication of the AP [20–22]. Although EN through the nasogastric route was the standard of care in most critically ill patients, NJT is widely needed in AP patients due to the systemic inflammation and local effusion accompanying the disease. Jejunal feeding is the most effective way to provide sufficient enteral nutrition in patients showing signs of intolerance to gastric feeding. It was reported that patients with SAP complicated by organ failure and/or pancreatic necrosis or fluid collections were at risk of gastric outlet obstruction, which could lead to aspiration and reflux; thus, they could probably benefit from nasojejunal feeding [23].

Our results showed that all patients had progressively increased calorie intake after NJT placement during the three following days, but the increment was more significant in the ultrasound group. However, the difference in the amount of delivered EN between the two groups may not be entirely attributed to the different insertion techniques. The organizational issues on the ward (delays to transfer the patients, delays to prescribe the EN, etc.) could also account for the observed difference. Besides, we found that ultrasound-assisted NJT placement at the bedside could significantly decrease the time required between prescription and initiation of NJT placement, as endoscopic procedures commonly require transferring patients to endoscopy suites and basic anesthesia, both of which are time-consuming.

Since intra-abdominal hypertension is a common and clinically relevant complication of AP [24–27], the fluctuation of IAP after NJT placement may impact the tolerance of EN, leading to delayed nutritional support [28–30]. Previous studies showed that increased IAP could cause a myriad of pathologic changes with a reduction in abdominal blood supply, including mesenteric vessels [31–33], and subsequently impairs intestinal barrier function and gastrointestinal motility owing to the ischemia of the gastric mucosa [34]. Our results showed a slight increased IAP after endoscopic procedures. On the contrary, the IAP in the ultrasound-assisted group showed a declining trend after NJT placement [35]. Although the difference is trivial and may not impact patient-centered outcomes, the ultrasound-assisted procedure is still worth recommending because of its convenience and excellent success rate.

There are some limitations in the present study that need to be discussed. First, as a retrospective study with a relatively small sample size, potentially confounding factors cannot be well controlled with randomization, and therefore, selection bias exists. In addition, the gastric residual volume and enteral tolerance were not continuously monitored during the study period, making the comparison less informative. Finally, we did not perform a cost-benefit analysis, which may be an additional advantage of ultrasound-assisted NJT placement over endoscopy.

## 5. Conclusion

This study shows that ultrasound-assisted NJT placement was comparable to endoscopic techniques with the acceptable success rates of the initial placement procedure in AP patients. However, its impact on clinical outcomes needs to be assessed in future randomized controlled studies.

## Figures and Tables

**Table 1:** Demographic and baseline characteristics.

	Ultrasound-assisted (*n* = 30)	Endoscopy (n =23)	*P*
Age (y)	47.2 ± 11.4	46.1 ± 12.3	0.61
Male, no. (%)	15 (50.0)	7 (69.6)	0.19
BMI (kg/m^2^)	26.6 ± 4.2	27.5 ± 3.6	0.38
APACHE II	12.5 (8-17)	11 (8-16)	0.63
Etiology, no. (%)			0.49
Biliary	14 (46.7)	15 (65.2)	
Hyperlipidemia	14 (46.7)	7 (30.4)	
Alcoholic	1 (3.3)	1 (4.3)	
Idiopathic	1 (3.3)	0	
NRS-2002 score at admission	3 (2-4)	2 (2-4)	0.28
CT severity index	6 (5-10)	8 (4-10)	0.87
SOFA at admission	5 (3-6)	3 (2-7)	0.15

Data are presented as *n* (%) or median (interquartile range). BMI: body mass index; APACHE II: Acute Physiology and Chronic Health Evaluation II; DBC: determinant-based classification; SOFA: sequential organ failure assessment.

**Table 2:** Clinical outcomes.

Variables	Ultrasound assistance (*n* = 30)	Endoscopy (*n* = 23)	*P*
Successful primary tube placement, no. (%)	28 (93.3%)	22 (95.7%)	1.00
Time between prescription and initiation of NJT placement (h)	4 (3-6)	12 (6-24)	<0.001
Procedural time (min)	20 (17.25-25)	20 (15-20)	0.02
Length of ICU stay (d)	8 (3.75-17.25)	8 (3-23)	0.73
Infected necrotizing pancreatitis, no. (%)	13 (43.3)	10 (43.5)	0.99
Mortality, no. (%)	5 (16.7)	6 (26.1)	0.62

Data are presented as *n* (%) or median (interquartile range). NJT: nasojejunal tube; ICU: intensive care unit.

## Data Availability

Data are available from the corresponding author on reasonable request.
